# Isolated ACTH deficiency following immunization with the BNT162b2 SARS-CoV-2 vaccine: a case report

**DOI:** 10.1186/s12902-022-01095-3

**Published:** 2022-07-19

**Authors:** Shuhei Morita, Tomoya Tsuji, Shohei Kishimoto, Shinsuke Uraki, Ken Takeshima, Hiroshi Iwakura, Hiroto Furuta, Masahiro Nishi, Hidefumi Inaba, Taka-aki Matsuoka

**Affiliations:** 1 First Department of Medicine, Wakayama Medical University, 811-1 Kimi-idera, Wakayama, 641-8509 Japan; 2 Department of Diabetes, Endocrinology, and Metabolism, Japanese Red Cross Wakayama Medical Center, Wakayama, Japan

**Keywords:** COVID-19, Vaccine, Isolated adrenocorticotropic hormone deficiency, Autoimmune/inflammatory syndrome induced by adjuvants (ASIA)

## Abstract

**Background:**

The global COVID-19 pandemic requires urgent development of new vaccines. Endocrinological adverse effects following the new mRNA vaccine against COVID-19 have been reported in several cases. Specific to the involvement of pituitary function; however, only a single case with hypophysis has been reported. This is the first case of isolated adrenocorticotropic hormone (ACTH) deficiency (IAD) following mRNA vaccination against COVID-19.

**Case presentation:**

A healthy 31-year-old man received the BNT162b2 SARS-CoV-2 mRNA vaccine. The first injection was uneventful. One day after the second injection, he noticed general fatigue and fever. In the following several days, he additionally developed headaches, nausea, and diarrhea. Four days after the vaccine injection, he visited a hospital with worsening of these symptoms. Physical examination revealed slight disorientation but no other deficits. Laboratory tests revealed hyponatremia, hypoglycemia, and extremely low plasma ACTH and serum cortisol levels (ACTH < 1.5 pg/ml, cortisol 1.6 μg/dl). He was diagnosed with adrenal crisis and was emergently treated with hydrocortisone. The symptoms responded well and he recovered within a few days. Magnetic resonance images after the replacement with hydrocortisone revealed an atrophic pituitary gland. The patient was referred to our tertiary hospital for further endocrinological examination. Pituitary endocrine load tests revealed isolated adrenocortical response deficiency. After other clinical assessments, he was diagnosed as having isolated ACTH deficiency. After initiation of hydrocortisone replacement, there has been no recurrence of symptoms related to adrenocortical insufficiency nor involvement of other pituitary functions.

**Conclusion:**

This is the first reported case of IAD potentially associated with COVID-19 immunization. Recent reports have emphasized the importance of adjuvants in the mRNA vaccine that induce the endocrinological adverse effects through disturbance of the autoimmune system, but details are still unclear. Given the broad and rapid spread of vaccinations against COVID-19, it is clinically important to consider that there could be cases with a rare but emergent adrenal crisis even among those who present common symptoms of adverse effects following inactive SARS-CoV-2 mRNA vaccination.

## Background

The rapid development of new vaccines has been urgently required in response to the global COVID-19 pandemic [1]. Besides common adverse effects, including several days of pain at the injection site, fatigue, headaches, and fever, there are also several reported cases of endocrinological effects that are potentially due to the new vaccines against COVID-19 [1]. Most of adverse endocrinological events are related to thyroid disorders [2–5], but one case of hypopituitarism has been potentially associated with COVID-19 immunization [6].

Isolated adrenocorticotropic hormone (ACTH) deficiency (IAD) is a rare disease in which ACTH secretion is specifically impaired; it can sometimes cause emergent events due to adrenal crisis. Acquired IAD is frequently accompanied by various autoimmune diseases [7, 8]. Furthermore, IAD has recently been focused as immune-related adverse events (irAE) due to the immune checkpoint drugs against PD1/PD-L1 antigen, additionally suggesting immune system disturbance in IAD [8, 9]. Based on these clinical findings with evidence from basic science, disturbance of specific autoimmunity system against corticotrophs is speculated to induce or develop pathogenesis of IAD [7, 8]. However, the precise mechanisms behind the development of autoimmunity against corticotrophs remains unknown.

Here, we present the first known case of IAD in which diagnosis was made following an adrenal crisis potentially related to the immunization with the BNT162b2 SARS-CoV-2 vaccine. Recent reports on endocrinological adverse effects from the vaccine have been emphasized the critical role of adjuvants within the vaccine in boosting adverse immune responses [2, 10], but further investigations are needed to elucidate if and how mRNA-based SARS-CoV-2 vaccine could cause ACTH-specific deficiency. It is clinically important to share the information that there could be cases with a rare but emergent adrenal crisis among patients with the symptoms of common adverse effects following the new vaccine.

## Case presentation

**Fig. 1:**
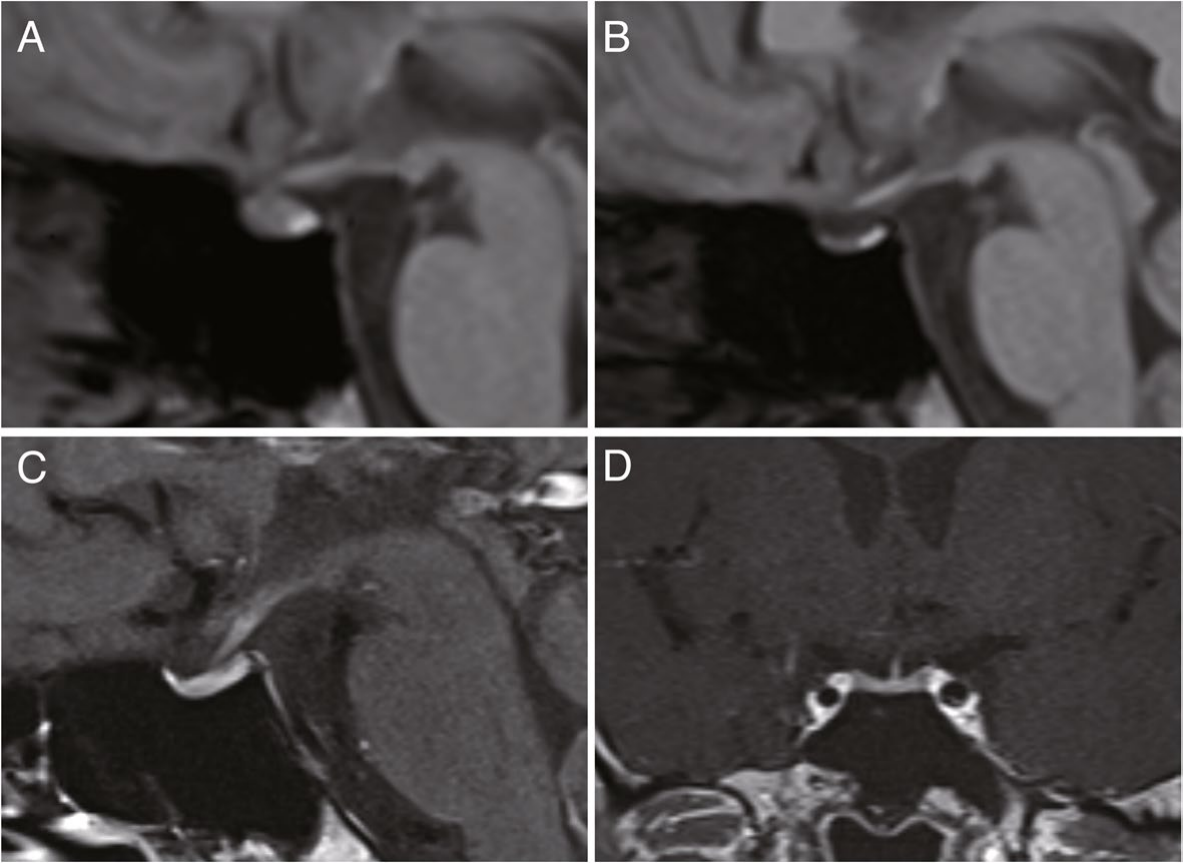
**A** T1-weighed pituitary MRI performed around two years before the onset. **B** T1-weighed pituitary MRI performed around two weeks after the replacement with hydrocortisone. **C**, **D** T1-weighed Gadolinium-enhanced pituitary MRI performed one month after the onset

A 31-year-old man with no significant medical history was in his usual state of health when he received his first dose of the BNT162b2 SARS-CoV-2 vaccine. Three weeks later, he received his second dose of the vaccine. One day after the second injection, he noticed general fatigue and fever, which remained for several days. The patient also developed headaches, nausea, and diarrhea over the next several days. Four days after the vaccine injection, he emergently visited a nearby hospital with these symptoms. On presentation, the pulse rate was 70/minute, blood pressure was 100/60 mmHg, and the body mass index was 19.4 kg/m^2^. There were no definite symptoms of postural hypotension. Physical examination revealed slight disorientation, but was other unremarkable and there were no neurological findings. The initial laboratory test revealed hyponatremia and hypoglycemia (Table 1), and low plasma ACTH and serum cortisol levels at 08:00 AM (ACTH < 1.5 pg/ml, reference range 8.7–61.5 pg/ml; cortisol 1.6 μg/dl, reference range 7.07–19.6 μg/dl at 06:00–10:00 AM), suggesting secondary adrenocortical deficiency. The cerebrospinal fluid examination was unremarkable and subsequent blood cultures were negative. Whole-body CT images including the brain showed no definite abnormality. The patient was diagnosed as having an adrenal crisis. Hydrocortisone (HC) was immediately replaced and his symptoms including slight disorientation recovered within several days. The dose of HC replacement was started from 200 mg/day, and it was gradually reduced to 15 mg/day in around two weeks. Magnetic resonance imaging (MRI) to investigate the pituitary gland revealed moderate atrophy of the anterior pituitary gland as compared with images taken two years previously, when there had been suspicion of cerebellar arteriovenous malformation (Fig. 1A and B). The high intensity of the posterior pituitary gland by the T1-weighted image was preserved (Fig. 1B). One week after the HC initiation, the patient was no longer symptomatic. The replacement with HC (15 mg/day) was continued.

**Table 1:** Laboratory data

		Reference range			Reference range
RBC (10^4/μL)	411	386–492	TSH (μU/mL)	8.470	0.61–4.23
WBC (10^4/μL)	69	33–86	Free-T3 (pg/mL)	2.49	2.30–4.00
Neutro (%)	54.9		Free-T4 (ng/dL)	1.22	0.90–1.70
Lymph (%)	34.1		Anti-TPO Ab (IU/mL)	< 3	0.0–2.9
Eosin (%)	1.4		Anti-Tg Ab (IU/mL)	< 5	0.0–4.9
PLT (10^4/μL)	15.6	15.8–34.8	Anti-TR Ab (IU/L)	< 0.9	< 2.0
Albumin (g/dL)	4.6	4.1–5.1	Prolactin (ng/mL)	21.4	
Creatinine (mg/dL)	0.60	0.65–1.07	ACTH (pg/mL)	6.9	8.7–61.5
eGFR (ml/min/1.73m^2^)	126.6		Cortisol (μg/dL)	1.6	
Sodium (mmol/L)	114.4	138–145	GH (ng/mL)	0.1	
Potassium (mmol/L)	3.6	3.6–4.8	IGF-1(ng/mL)	128	107–297
Chloride (mmol/L)	84.3	101–108	LH (mIU/mL)	3.8	
Blood glucose (mg/dL)	39	73–109	FSH (mIU/mL)	2.9	
IRI (μU/mL)	< 0.2	2.1–19.0	ADH (pg/mL)	1.2	
CPR (ng/mL)	0.1	0.74–3.18			
AST (U/L)	87	13–30			
ALT (U/L)	28	7–23			
LD_IFCC (U/L)	343	124–222			
γ-GTP (U/L)	10	9–32			

Left column: Blood count and biochemistry tests were performed at the first admission in the previous hospital. Right column: Basal endocrinological tests were performed in our hospital

**Fig. 2:**
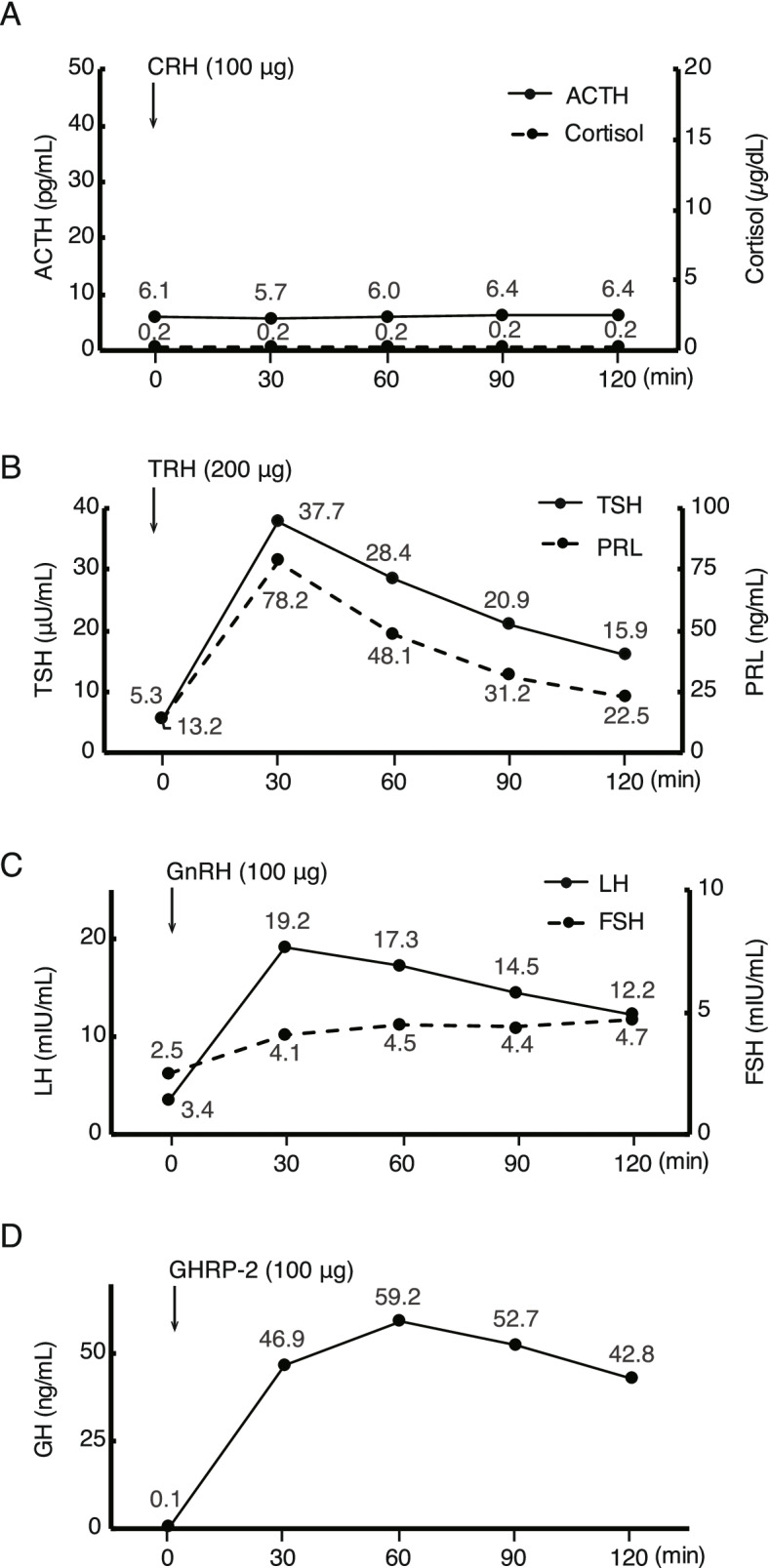
Pituitary provocation test one month after the onset. **A**, **B**, **C**, and **D** represent the results after CRH (100 μg, i.v.), TRH (200 μg, i.v.), GnRH (100 μg, i.v.), and GHRP-2 (100 μg, i.v.) load, respectively

One month later, the patient was admitted to our tertiary hospital for further endocrinological examinations. Physical examination was unremarkable. A basal endocrinological test revealed low plasma ACTH (6.9 pg/ml) and cortisol (1.6 μg/dl) levels (Table 1), which confirmed the results from the previous hospital. To further investigate the pituitary function, CRH (100 μg), TRH (200 μg), GnRH (100 μg) and GHRP-2 (100 μg) load tests were performed (Fig. 2) in the previously described manner [11–14] to confirm normality of not only basal, but also responsive secretion of pituitary hormones other than ACTH [11]. The adrenal response was markedly abnormal; the low baseline cortisol level of 0.2 μg/dl stayed at the same level at all the time points up to 120 min. The baseline ACTH level was also low at 6.1 pg/ml and remained low for up to 120 min, indicating secondary adrenal insufficiency (Fig. 2A). On the other hand, TSH, LH/FSH and GH showed normal reactions after TRH, GnRH and GHRP-2 load, respectively (Fig. 2B-D). MRI images with gadolinium-enhancement revealed an atrophic pituitary gland, as had been shown in plain MRI images in the previous hospital, but no remarkable enhanced lesion (Fig. 1C, D). Together, the pituitary endocrine test with other laboratory tests revealed isolated ACTH insufficiency. Paraneoplastic mechanisms have been shown to be a cause of IAD [14], so chest and abdominal CT scans were performed; there were no malignant lesions including thymoma. Increased basal TSH and normal free T3 and T4 levels suggested subclinical hypothyroidism (Table 1). Thyroid sonography was normal and thyroid autoimmune antibodies were within the normal range (Table 1). Three months after initiation of HC replacement, there has been no recurrence of symptoms with adrenocortical insufficiency or involvement of other pituitary functions. As for hypothyroidism, although the etiology was unknown, continuous evaluation was planned if it was transient or persistent at the regular outpatient clinic.

## Discussion and conclusions

This is the first case of IAD following immunization with the BNT162b2 SARS-CoV-2 vaccine. The first workup at the previous hospital revealed central adrenocortical insufficiency, and immediate replacement with HC was initiated. Further endocrinological investigation at our tertiary hospital confirmed the presence of IAD.

After the first Food and Drug Administration (FDA) approval of a vaccine against the SARS-CoV-2 virus in December in 2020, several adverse effects have been reported, including endocrinological impairments [1, 4, 6]. Among them, thyroid disorders are the most commonly reported adverse endocrinological events potentially due to the new vaccine. To date, Graves’ disease, silent thyroiditis, and subacute thyroiditis have been reported as side effects [2–5]. Based on the inclusion of adjuvants in the new vaccines and their important role in immune response, those thyroid diseases following the vaccine are, at least in part, thought to be a part of the autoimmune/inflammatory syndrome induced by adjuvants (ASIA) [2, 10]. ASIA is an autoimmune and/or inflammatory syndrome induced by adjuvants and develops through disruption of the host’s immunological balance by molecular mimicry, triggering polyclonal activation of B lymphocytes, or other similar pathogenetic mechanisms in susceptible individuals. Similar to thyroidal adverse effects, our case also meets the diagnostic criteria of ASIA defined by Shoenfeld et al. in that no symptoms were shown in relation to adrenal insufficiency before vaccination, and there was the presentation of fever and chronic fatigue [10]. It remains unknown if and how the vaccine specifically affected corticotrophs, although it could be a trigger for induction or manifestation of IAD to activate autoimmune cascades and pathways by adjuvants, leading to impairment/disturbance of the host’s immunological balance.

Although it is difficult to confirm if and how the SARS-CoV-2 mRNA vaccine induces IAD, we speculate on the role of the vaccine. One possibility is that the vaccine either primarily or secondarily caused the impairment of corticotrophs to induce acute adrenal insufficiency. As the serum cortisol level at the first detection and 24 h urinary free cortisol levels were remarkably low (4.4 μg/day), it is assumed that adrenal insufficiency is acute and severe enough to be unlikely to survive without presenting symptoms for a long time. Furthermore, as discussed above, recent evidence highlighted potential problems associated with adjuvants in the SARS-CoV-2 mRNA vaccine [2, 10].

Another possibility is that common adverse effects from the vaccine increased adrenocortical demand [15], which led to the secondary subclinical adrenal deficiency. In any case, since similar reports related to hypophysis following the vaccine are limited, it is important to share that the vaccines could be associated with rare but serious adverse effects so that there can be rapid identification and early therapeutic initiation.

Another feature of our case is that the MRI images showed atrophy of the pituitary gland compared to the images taken two years ago. We speculate that this could be because the images were taken after the replacement with HC, so the pituitary gland showed a good response in radiology. In the only known reported case with hypophysis following COVID-19 vaccine, there was a rapid response to HC replacement within a month, showing diminished enlarged gland like a mostly empty sella, which was similar to the current case [6].

Pituitary MRI findings of IAD are vary depending on the etiology. For example, IAD related to the anti-PIT1 hypophysitis was reported to be slightly atrophic or normal on pituitary MRI [14]. On the other hand, IAD related to ICIs, especially anti-PD-1/PD-L1 inhibitors, could cause isolated adrenal insufficiency without enlargement of the pituitary gland and stalk [8, 9].

Regarding the functional aspect, given the quick response shown in pituitary imaging, the replacement might have been initiated rapidly enough to spare pituitary function other than the first involvement of the adrenocortical system. Early detection and rapid initiation with HC replacement may have a critical role in sparing other pituitary functions, especially in the specific situation of hypophysis.

In conclusion, this is the first case of IAD potentially associated with COVID-19 immunization. Given the autoimmune aspect of IAD and the increased possibility of adversely affecting the autoimmune systems via the adjuvants in the vaccine, IAD could be associated with inactive SARS-CoV-2 mRNA vaccination. As consideration for early detection and treatment, there may be cases with a rare but emergent adrenal crisis, even among those presenting common symptoms of adverse effects following the SARS-CoV-2 mRNA vaccination.

## Data Availability

The datasets used and analyzed during the current study is available from the corresponding author on reasonable request.

## Abbreviations

ACTH: Adrenocorticotropic hormone
ASIA: Autoimmune/inflammatory syndrome induced by adjuvants
FDA: Food and Drug Administration
HC: Hydrocortisone
IAD: Isolated adrenocorticotropic hormone deficiency
irAE: Immune-related adverse event
MRI: Magnetic resonance imaging
